# Precision Medicine in Inflammatory Bowel Disease

**DOI:** 10.3390/diagnostics13172797

**Published:** 2023-08-29

**Authors:** Vito Annese, Monica Annese

**Affiliations:** 1 Department Gastroenterology IRCCS San Donato Policlinic, Vita-Salute San Raffaele University, 20100 Milan, Italy; 2 Department Gastroenterology, IRCCS Hospital Casa Sollievo della Sofferenza, 71013 San Giovanni Rotondo, Italy

**Keywords:** inflammatory bowel disease (IBD), ulcerative colitis (UC), Crohn’s disease (CD), precision medicine (PM), artificial intelligence (AI)

## Abstract

Ulcerative colitis and Crohn’s disease are traditionally defined as the two main subtypes of inflammatory bowel disease. However, a more recent view considers IBD as a spectrum of heterogeneous phenotypes with consistent differences in clinical presentation and behaviors, likely explained by differences in underlying pathogenetic mechanisms. The etiology is still elusive, and the suggested pathogenesis is a complex interplay among genetic predisposition and abnormal immune response at the mucosal intestinal level, activated by only partially identified environmental triggers leading to altered intestinal permeability and impaired handling of gut microbiota. The undeniable continuous progress of medical therapy with more frequent shifts from traditional to more advanced modalities also underlines the actual unmet needs. We are using medications with completely different mechanisms of action, with a lack of predictive factors of outcomes and response and still an unsatisfactory rate of success. In addition, we are missing still valuable and accurate markers to predict disease progression and severity in order to avoid under- or over-treatment. In such a complex scenario, it is undoubtful that the application of artificial intelligence and machine learning algorithms may improve the management and pave the way for precision and eventually personalized medicine in these patients; however, there are still several challenges that will be the focus of this review.

## 1. Introduction

### A Look at the Complexity of IBD

Inflammatory bowel diseases (IBDs) are progressively increasing their prevalence worldwide with over 2 million North Americans and 2 million Europeans currently diagnosed. Although the incidence has almost plateaued in these countries, it is steadily increasing elsewhere with probably 6 million affected globally [1]. Therefore, a progressive increase in the burden for society with massive pressure on health care costs worldwide is expected. The clinical variability of behaviors is striking with cases with limited involvement and a favorable course and probably the majority with progressive bowel damage and complications leading to hospitalization, surgery and eventually cancer [2,3]. In addition, up to half of patients may have extraintestinal manifestation, and as a whole, all have an increased risk for other immune-mediated diseases. It is frustrating to consider that still the possibility to prognosticate the disease course is based on rather few and yet not solid and accurate clinical features (Table 1—modified from [4]), lacking appropriate biomarkers.

Although the therapeutic armamentarium is continuously expanding, approximately one-third of the patients are primary non-responders to the initial treatment, and up to half will have incomplete or a loss of response over time. In addition, we are using medications with completely different mechanisms of action, with a lack of predictive factors of outcomes, response and tolerability.

In this scenario, it has been hypothesized that given the failure of traditional statistical methodologies in improving the prediction of outcome, response to the therapy, etc., the application of different approaches such as machine learning (ML) and artificial intelligence (AI), by pulling and analyzing a large amount of clinical and biological data, might become a breakthrough and a game changer in IBD as is happening in oncology. In the next sections, the concept of precision medicine will be clarified, as well as the actual impact and application of the new methodologies and analysis in IBD and the different challenges to overcome. A more extensive review has been recently conducted in a Scientific Workshop of the ECCO [5,6].

## 2. The Potential of Precision Medicine IBD

The concept of precision medicine is not new and was first reported in a publication in 1971 [7]. However, since then, many other terminologies have been used and eventually not with the same meaning, such as “individualized medicine”, “personalized medicine”, “tailored medicine”, etc. Of note, the US National Council Committee [8] stated that the term “precision medicine” should be used for the “*tailoring of medical treatment to the individual characteristics of each patient to classify individual in subpopulations that differ in their susceptibility to a particular disease or their response to a specific treatment*”. In addition, the Committee clearly stated the distinction between “precise” and “personalized” medicine by clarifying that “*personalized medicine refers to treatment tailored towards single individual*” while “*precision medicine seeks to identify homogenous subgroups stratified according to a similar behavior*”.

**Figure 1:**
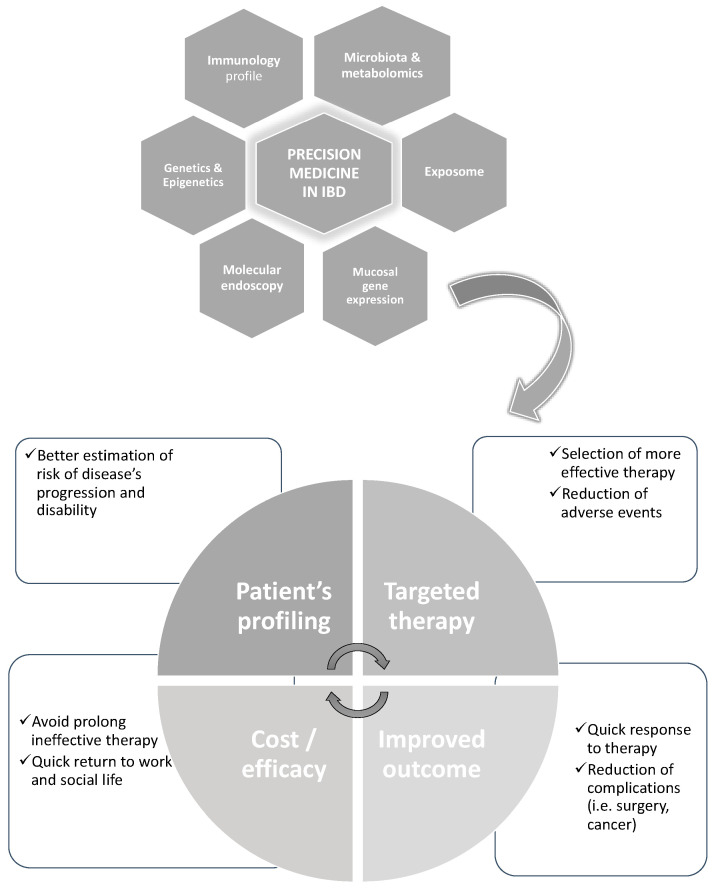
An example of the potential of the evaluation by system biology of multi-omics in IBD aiming to achieve with more “precision” a tailored therapy and better outcomes.

More recently, another publication [9] provided a more extensive and modern view stating that “PM seeks to improve stratification and timing of health care by utilizing biological information and biomarkers at the level of molecular disease pathways, genetics, proteomics as well as metabolomics”—essentially, embracing the concept of the application of system biology and bioinformatics in health care. System biology allows the possibility of analyzing the interaction of all the components in a well-defined biological context [10]. In the scenario of IBD, this means analyzing the role of environmental factors (so-called exposome), predisposing genetic factors (genome), the possible non-genetic modification of the genome (epigenome) and gene expression at the mucosal levels including coding and non-coding RNAs (transcriptome and proteome), as well as the differences in gut microbiota and their produced metabolites (microbiome and metabolome), all at once (Figure 1). The analysis of these omes and omics may be achieved only by bioinformatics [11] with methodologies still under continuous evolution.

## 3. The Potential of Artificial Intelligence in IBD

Any attempt for a single investigator to analyze all the possible information related to the “omics” of a single patient with IBD, resulting in millions of data points, is obviously grossly inadequate. Rather, AI and its computational revolution and speed may support and unravel the underlying complexity of such biology. This may likely be achieved with the use of ML, essentially, a branch of AI in which computer algorithms improve automatically through the experience, in a hypothesis-free context [12].

**Figure 2:**
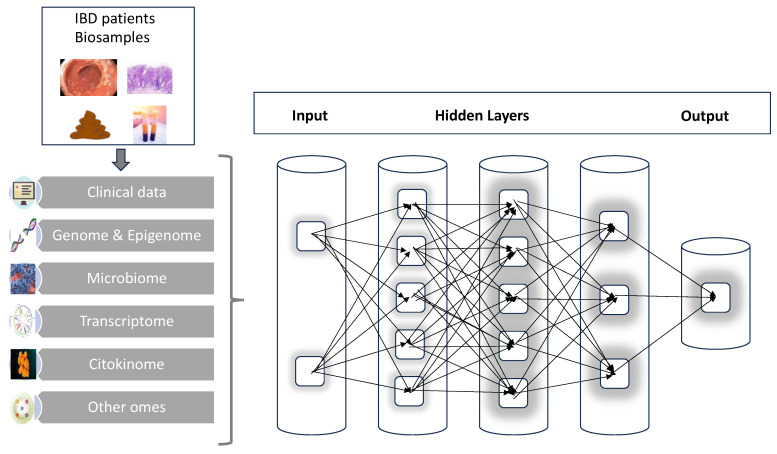
A graphic scheme of the multi-step process of analysis starting from patient’s biosamples; the combination of clinical data and multi-omics results in artificial neural networks will progressively elaborate the large complexity and number of data to deliver hopefully more individualized and precise output.

Conceptually, a possible structure for an IBD neural network can be organized into three major layers. The first layer is where all the input is conveyed, responsible for handling the individual IBD omics such as genome, transcriptome, etc. Next, we must imagine hidden layers that will perform all the complex calculations, interactions and combinations of parameters. Finally, there will be the output layer that, after receiving the signals for the hidden layers, will finally process and provide the results (Figure 2). There are already preliminary applications and results of this methodology that will be detailed in the next sections. There is, however, still the need to evaluate in IBD which is the best computation tool for the specific information that is under investigation.

## 4. Initial Applications in IBD

### 4.1. Prediction of Disease Course

Given the great heterogeneity, it is very likely that understanding the disease course and evolution is one of the cornerstones for developing a precision medicine approach to IBD. Traditionally, some clinical features have been utilized as depicted in Table 1, but they either are not validated or lack sufficient prognostic accuracy.

An emerging area is the comprehensive evaluation of intestinal involvement in CD not only with endoscopy but utilizing imaging-based biomarkers. More specifically, one potential tool is the so-called Lemann Index (LI) which is a scoring system that combines the utilization of clinical and endoscopic figures with imaging obtained with magnetic resonance enterography (MRE). The evaluation of bowel damage already at the diagnosis might help decision making in favor of more aggressive therapy, and periodic re-evaluation might estimate the increase or reduced disease burden [13]. More importantly, the evaluation of bowel damage has been demonstrated as an independent prognostic risk factor for intestinal surgery (Hazard Ratio (HR) = 3.2) and hospitalization (HR = 1.9) [14].

Another important contribution has been achieved by the pediatric cohort of the RISK study [15] which has demonstrated that combining the clinical information (age, race, disease location), antimicrobial serologies and gene signature from ileal biopsies (more specifically, an extracellular matrix signature) with a system biology methodology has been able to predict the more aggressive course of the disease such as the development of stricturing disease in 3 years of disease course. The study in addition has underlined the importance of the prospective validation of the supposed biomarkers in the same individuals. In contrast, very frequently, many described biomarkers have failed replication on independent cohorts because of large disease heterogeneity.

Because of the invasiveness of the endoscopic procedure and the restriction for more frequent re-evaluation, the focus has been shifted to the utilization of blood-based biomarkers. Unfortunately, neither the investigation of DNA methylation [16] nor the genomewide association studies (GWAs) [17] have demonstrated adequate power to identify variants with an elevated odds ratio (OR) which is useful to direct patient stratification. In CD, *NOD2* gene polymorphism has been associated with ileal localization, younger age and a more complicated disease course [18], while the risk allele of the *ATG16L1* gene has been associated with perianal disease [19]. In UC, the HLA-DRB1*0103 allele has been associated with an increased risk of pancolitis, extra-intestinal manifestation and need for colectomy [20]. In addition, HLA-B27-positive IBD patients have a higher risk of ankylosing spondylitis [21].

A significant contribution has been the discovery by Lee, J.C. et al. [22] of a validated prognostic test derived from the gene-expression signature of CD8+ T cells which predicted a 3-fold increase in the need to escalate the therapy or need for surgery in Crohn’s disease. Accordingly, only very recently [23], this biomarker has been applied in the PROFILE trial to stratify patients with CD to different treatments, and the results are still awaited. This also underlines the time lag from discovery and validation to utilization and eventually translation in clinical practice of the biomarkers.

Another easily accessible biomarker, but not less complex to analyze, is the microbiota. There are some reports such as the reduction in *Faecalibacterium Prausnitzii* [24] in the resected ileum that might predict a more frequent relapse or a specific transcriptome [25] and microbiome signature at diagnosis in pediatric Crohn’s predicting a higher rate of steroid free remission, but further validations are awaited.

### 4.2. Prediction of Complication of Therapy

Genotyping of the thiopurine S-methyltransferase (*TMPT*) gene has been largely used to predict the possible bone marrow toxicity of thiopurines, determine the correct dosage [26] and reduce toxicity. More recently, the polymorphism of the *NUDT15* gene has been identified as a major risk factor for the bone marrow toxicity of thiopurines [27]. The combined polymorphism of these two genes explained almost 50% of the thiopurines-induced myelotoxicity. The polymorphism of the HLA-DQA1*02:01-HLA-DRB1*07:01 haplotype [28] has been identified as predictive of thiopurine-induced pancreatitis, but given the rarity (number needed to test = 76), testing is limited in clinical practice. In addition, the HLA- DRB1*03:01 allele has been demonstrated to increase the risk of 5-ASA-induced nephrotoxicity [29] while the HLA-DQA1*05 variant [30] doubles the risk of the development of antibodies to both infliximab and adalimumab. Interestingly, a prospective trial is ongoing in Canada (NCT04109300) [31] to evaluate the clinical utility of this genetic testing before the initiation of therapy with anti-TNF agents.

Patients with an *NAT2* gene polymorphism, particularly the slow metabolizer, have a risk of developing sulphapyridine-induced adverse events when using salazopyrin [32]. The presence of the methylenetetrahydrofolate reductase (*MTHFR*) 1298C variant has been associated with a higher risk of side effects in IBD patients using methotrexate [33]. The carriage of the minor risk allele of the *FASLG* gene (rs76110) has been demonstrated to increase the risk of severe infusion reactions to infliximab [34].

Actually, the production of a chip with polymorphism-based genetic testing is quite simple and might help to identify patients at risk of side effects before the treatment, but cost–benefit analysis studies are still lacking.

### 4.3. Prediction of Response to Therapy

Given the substantial rate of primary non-response of any therapy for IBD as well as a lack of complete response or a loss of response, the possibility to predict the efficacy of the therapy and hence more precise tailoring of the management for every patient is probably the most important unmet need.

One fascinating option is the so-called “*molecular endoscopy*”. Essentially, during the endoscopy, an anti-TNF fluorescent antibody liquid can be sprayed. Subsequently, with confocal laser endoscopy, it is possible to quantify the cells showing mTNF. By using this methodology, Atreya et al. [35] demonstrated a 92% response to anti-TNF therapy after 3 months in a patient with CD with elevated mTNF expression; in contrast, the response to the therapy was only 13% in a patient with low mTNF expression. More importantly, the efficacy of the therapy was retained at 1 year of follow-up. The same group with a similar methodology demonstrated a higher efficacy of therapy with vedolizumab in Crohn’s disease in patients with enhanced expression of α4β7 integrin [36].

The evaluation of *genetic polymorphisms* in IBD has been particularly successful in identifying over 250 susceptibility markers of disease risk, but it is not very informative toward the prediction of the efficacy of therapy. More data have been obtained but often discordant about the response to anti-TNF therapy. Patients with UC homozygous carriers of the high-risk variants of the *IL-23R* gene have displayed a better response compared to patients with carriers of the low-risk variants [37]. In a Belgian cohort [38], patients with CD with Fas ligand -843 CC or the CT genotype had a higher response to infliximab compared to patients with carriers of the TT genotype. The presence of a homozygous variant of the IBD5 locus has been associated with non-response to infliximab in CD but not in UC [39].

An alternative approach in the attempt to predict the response to the therapy has been the evaluation of the *immunologic profile*. The higher serum level of IL-23 in patients with CD did predict a better response to MEDI 2070, a selective agent against IL-23, but definitively more confirmations are needed [40].

In IBD, there is definitely a change in the microbiota with reduced diversity and different abundances of some species; whether it is a primary or secondary phenomenon is still unclear [24]. What is intriguing is the concept that differences in microbiota might also influence the response to the therapy [41]. In general, patients with a more diverse baseline microbiome and higher microbial diversity have shown a better response to anti-TNF therapy but also vedolizumab and ustekinumab. A better response was seen in the presence of fewer mucus-colonizing bacteria, a lower abundance of pro-inflammatory strains and a higher abundance of short fatty acid-producing strains [42]. One study has demonstrated a better efficacy of vedolizumab in CD patients with a higher degree of α-diversity [43].

Many studies have focused on the change in *gene expression* at a mucosal level as a possible marker for the prediction of response to the therapy. Arijs et al. [44] for the first time analyzed the gene expression profile of the colon in a patient with ulcerative colitis. Initially, they used a probe with 212 gene markers, and subsequently, they selected the top 5 with higher differences in expression; with this methodology, they were able to predict the efficacy of infliximab with 96% sensibility and 85% specificity. The same group replicated the experience in colonic CD by selecting four of the identified genes in the study of UC (*IL13RA2*, *IL-11*, *IL-6*, *TNFAIP6*) [45]. Unfortunately, the same signature could not be replicated using samples of the ACT1 trial [46] and a phase 2A trial with golimumab [47]. In another experience [48], the serum level of Oncostatin M, its receptor and transcript in the inflamed mucosa did correlate with failure to respond to infliximab. In addition, Oncostatin M itself was hypothesized to also have a pathogenetic role. Gaujoux et al. [49] by evaluating the mucosa expression profile demonstrated a correlation between the amount of plasma cells and the expression of *CCL7-CCR2* and *TREM1* genes and response to the therapy. More recently, the response to etrolizumab [50], an anti-β7 integrin, was enhanced in patients with UC and increased mucosal levels of granzyme A and integrin αE. Baseline expression levels of four genes (*PIWILI*, *MAATSI*, *RGS13*, *DCHS2*) were able to predict endoscopic response to vedolizumab [51].

Conceptually, as earlier mentioned, the combination of more biomarkers with more sophisticated statistics could be in the future more productive. Some experiences are already accumulating. Barber et al. [52], by utilizing a genetic score based on the Illumina Immunochip, did predict with higher accuracy compared to clinical parameters the response to anti-TNF. In addition, 16 polymorphisms did predict a more prolonged efficacy of the therapy. Similarly in the previously mentioned RISK study [15], the combination of the information about fecal microbiota, the genetic profile and the presence of anti-microbic antibodies not only predicted a different course of the disease but also a better response to infliximab in some patient clusters. In an inception cohort of pediatric UC patients, the combination of the mucosal expression profile and an abundance of microbiota species in combination with the clinical index did predict upfront the patient requiring escalation therapy to anti-TNF [53]. A summary of these experiences is depicted in Table 2.

More information will be also achieved in the future from ongoing prospective studies such as the trial PROFILE (ISRCTN11808228) using a top-down vs. step-up approach of therapy based on the immunologic profile [23], as well as the study PREDICCT (ISRCNT67248113) [54] looking at the “prognostic effects of environmental factors in Crohn’s and Colitis” that will monitor the activity and outcome of the disease, taking into consideration also the diet, lifestyle and microbiota.

## 5. Future Directions and Conclusions

One of the challenges so far in the direction of precision medicine in IBD is that, unfortunately, some of the potential biomarkers found in one cohort have not been replicated in others. This may reflect different patient selection or simply be explained by the conclusion that some biomarkers are only associated with the response to the treatment but not relevant to its prediction. Therefore, still, much must be done in carefully designed prospective trials and well-selected populations to validate potential biomarkers before their introduction into clinical practice. Another important challenge is related to the high heterogeneity of IBDs and their modifications over time; therefore, large cohorts with adequate prolonged prospective evaluation will be required to achieve relevant information for daily clinical practice. Some of these cohorts have already been recruited (PANTS, IBD Resource, RISK, PROFILE), and others are ongoing (COLLIBRI, PREDICCT, IBD Multiomics) and will surely produce more information in the near future [55].

There are, however, even more gaps to close. The physicians should agree upon well-defined targets of outcome that are easily assessable, reproducible and relevant (i.e., endoscopic healing). A large cooperation will be needed to assemble large and well-characterized cohorts and to conduct adequately prolonged follow-up with adequate (randomized) study design. This will also require agreement on standardized treatment protocols. After that, replication studies with independent cohorts will be needed.

Next, there will be other possible limitations, such as making multi-omics datasets publicly accessible, clearly distinguishing cause and consequence and translating complex signatures to a likely non-invasive test easily used in daily clinical practice. Not to be forgotten that prospective multi-omics studies may have prohibitive costs.

The progressive increase in the prevalence and burden of the cost of IBD should prompt an alliance among academics, the industry and third parties to provide finance, infrastructure and opportunity to translate progressively the results from the laboratory and clinical studies to daily practice. It is time to change the strategy in the management of IBD from “reactive”, driven by the activity of the disease, flares and complications, to “proactive”, focusing on modifying the disease course and consequences first in a homogenous subgroup of patients with better-defined phenotypes and next in a single individual.

## Figures and Tables

**Table 1:** Example of clinical features suggesting a more aggressive course of IBD (modified from ref. [4]).

**Crohn’s Disease**	Age at diagnosis less than 40 years
	Use of systemic steroids at diagnosis
	Perianal involvement
	Ileo-colic localization
	Deep ulcers
	More extensive involvement
	Stenosing/Fistulizing behavior
**Ulcerative Colitis**	Younger age at diagnosis
	Female gender
	Extensive colitis
	Non-smoker

**Table 2:** Summary of the main experiences in IBD to predict the outcome of disease and therapy.

Marker	Target	Result	Reference
Disease Course			
Lemann Index	Bowel damage with endoscopy and MRE in CD	Increased risk for surgery and hospitalization	[13,14]
Genetic markers + antimicrobial antibodies + clinical parameters	Disease progression CD	More stricturing disease in 3 yrs	[15]
NOD2	Disease course CD	Stenosis, surgery	[18]
ATG16L1	Disease course CD	Perianal disease	[19]
HLA-DRB1*0103	Disease course UC	Pancolitis, EIMs, colectomy	[20]
HLA-B27	Disease course IBD	Increased risk of ankylosing spondylitis	[21]
CD8+ T cell signature	Disease course CD	More aggressive disease course	[22]
Faecalibacterium Prausnitzii	Disease course CD	More frequent relapse after surgery	[24]
Toxicity/Efficacy Therapy			
TPMT/NUDT15	Toxicity thiopurine	Explain 50% risk of myelotoxicity	[26,27]
HLA-DQA1*02:01-HLA-DRB1*07:01 haplotype	Toxicity thiopurine	Thiopurine-induced pancreatitis	[28]
HLA- DRB1*03:01	Toxicity of mesalamine	Increased risk of nephrotoxicity	[29]
HLA-DQA1*05	Immunogenicity anti-TNF	Double the risk of antibodies	[30]
NAT2 slow metabolizer	Toxicity of salazopyrin	Increased risk	[32]
MTHFR 1298C	Toxicity of methotrexate	Reduced tolerability	[33]
FASLG gene (rs76110)	Tolerability Infliximab	More frequent infusion reaction	[34]
mTNF expression at confocal laser endoscopy	Efficacy infliximab in CD	Correlated to mTNF level	[35]
α4β7 integrin expression at confocal laser endoscopy	Efficacy of vedolizumab in CD	Correlated to mucosal density	[36]
homozygous carrier of the high-risk variants of IL-23R	Efficacy of infliximab in UC	Increased	[37]
Fas ligand -843 CC or CT	Efficacy of infliximab in CD	Increased	[38]
Homozygous variant of IBD5 locus	Efficacy of infliximab in CD	Primary failure	[39]
Serum level IL-23	Response to MEDI 2070	Response correlated to serum level	[40]
Mucosal gene signatures	Response to infliximab in UC	Increased	[44]
IL13RA2, IL-11, IL-6, TNFAIP6	Response to infliximab in CD	Increased	[45]
Oncostatin M serum, receptor and transcript	Response to infliximab	Correlated	[48]
CCL7-CCR2 and TREM1 genes	Response to therapy	Correlated	[49]
Mucosal level of granzyme A and integrin αE	Response to etrolizumab UC	Correlated	[50]
PIWILI, MAATSI, RGS13, DCHS2 expression	Response to vedolizumab	Increased	[51]
Genetic Score with Immunochip Illumina	Response to infliximab	Predicted higher response	[52]
Microbiota + genetic profile + antimicrobial antibodies	Response to infliximab	Higher	[15]
Microbiota + expression profile + abundant microbiota + clinical index	Response to anti-TNF	Good prediction	[53]

## Data Availability

Not applicable.
